# Sub-Millisecond Laser-Irradiation-Mediated Surface Restructure Boosts the CO Production Yield of Cobalt Oxide Supported Pd Nanoparticles

**DOI:** 10.3390/nano13111801

**Published:** 2023-06-05

**Authors:** Praveen Kumar Saravanan, Dinesh Bhalothia, Guo-Heng Huang, Amisha Beniwal, Mingxing Cheng, Yu-Chieh Chao, Ming-Wei Lin, Po-Chun Chen, Tsan-Yao Chen

**Affiliations:** 1 Department of Materials and Mineral Resources Engineering, National Taipei University of Technology, Taipei 10608, Taiwan; spjp2766@gmail.com; 2 Department of Engineering and System Science, National Tsing Hua University, Hsinchu 30013, Taiwan; colorful032@gmail.com (G.-H.H.); amishabeni0607@gmail.com (A.B.); mingxing.cheng@liverpool.ac.uk (M.C.); bettyhome2007@gmail.com (Y.-C.C.); mwlin@mx.nthu.edu.tw (M.-W.L.); 3 Department of Electrical Engineering and Electronics, University of Liverpool, Liverpool L69 3GJ, UK; 4 Institute of Nuclear Engineering and Science, National Tsing Hua University, Hsinchu 30013, Taiwan; 5 Hierarchical Green-Energy Materials (Hi-GEM) Research Centre, National Cheng Kung University, Tainan 70101, Taiwan

**Keywords:** RWGS reaction, sub-millisecond laser, Pd nanoparticles, surface restructure, CO_2_ conversion

## Abstract

The catalytic conversion of CO_2_ into valuable commodities has the potential to balance ongoing energy and environmental issues. To this end, the reverse water–gas shift (RWGS) reaction is a key process that converts CO_2_ into CO for various industrial processes. However, the competitive CO_2_ methanation reaction severely limits the CO production yield; therefore, a highly CO-selective catalyst is needed. To address this issue, we have developed a bimetallic nanocatalyst comprising Pd nanoparticles on the cobalt oxide support (denoted as CoPd) via a wet chemical reduction method. Furthermore, the as-prepared CoPd nanocatalyst was exposed to sub-millisecond laser irradiation with per-pulse energies of 1 mJ (denoted as CoPd-1) and 10 mJ (denoted as CoPd-10) for a fixed duration of 10 s to optimize the catalytic activity and selectivity. For the optimum case, the CoPd-10 nanocatalyst exhibited the highest CO production yield of ∼1667 μmol g^−1^_catalyst_, with a CO selectivity of ∼88% at a temperature of 573 K, which is a 41% improvement over pristine CoPd (~976 μmol g^−1^_catalyst_). The in-depth analysis of structural characterizations along with gas chromatography (GC) and electrochemical analysis suggested that such a high catalytic activity and selectivity of the CoPd-10 nanocatalyst originated from the sub-millisecond laser-irradiation-assisted facile surface restructure of cobalt oxide supported Pd nanoparticles, where atomic CoOx species were observed in the defect sites of the Pd nanoparticles. Such an atomic manipulation led to the formation of heteroatomic reaction sites, where atomic CoOx species and adjacent Pd domains, respectively, promoted the CO_2_ activation and H_2_ splitting steps. In addition, the cobalt oxide support helped to donate electrons to Pd, thereby enhancing its ability of H_2_ splitting. These results provide a strong foundation to use sub-millisecond laser irradiation for catalytic applications.

## 1. Introduction

The thriving anthropologic activities result in increased atmospheric carbon dioxide (CO_2_) levels, which, consequently, has led to detrimental effects on the environment [1]. Therefore, establishing efficient CO_2_ utilization strategies is imperative. In this context, the catalytic transformation of carbon dioxide (CO_2_) into beneficial fuels and commodities is of potential interest [2,3]. Such an approach not only mitigates atmospheric CO_2_ concentration but also circumvents energy issues. Given that CO_2_ can be utilized as a C_1_ feedstock for producing various fuels, such as carbon monoxide (CO), methane (CH_4_), and formic acid (HCOOH), the selective CO_2_ hydrogenation (reduction/conversion) to CO via the reverse water–gas shift (RWGS) reaction is the most promising path because of its well-known industrial applications [4,5,6]. However, the competitive CO_2_ methanation (where the CO is further hydrogenated to produce methane (CH_4_)) severely hampers the rate of RWGS reaction, thereby suppressing the production yield of CO [7]. In addition, because of the endothermic behavior of the RWGS reaction, the exothermic CO_2_ methanation reaction is more favourable at low temperatures [8]. Therefore, it can be concluded that the development of highly active and selective catalysts for an RWGS reaction that can be operated at low temperatures is an urgent need for establishing a zero-carbon economy.

The RWGS reaction follows two main possible pathways: the redox and the associative pathways [9,10]. For the redox pathway, the CO_2_ molecule first dissociates into *CO and *O intermediates. There are two possibilities in this step: (i) the catalyst being oxidized due to *O atoms from the dissociation of the CO_2_ molecule or (ii) the CO_2_ dissociates at the oxygen vacancy (O^V^) sites to produce *CO. On the basis of the CO_2_ dissociation mechanism, the hydrogen (H_2_) molecule can reduce the catalyst or create oxygen vacancies (H_2_ + M-O → H_2_O + M; here, M is the notation for metal). Mostly, the catalytic materials comprising reducible oxides follow the redox pathway for completing the RWGS reaction. [11] In contrast, the associative mechanism follows a completely different pathway, where the H_2_ molecule first dissociates into *H atoms, which subsequently react with adsorbed *CO_2_ molecules to form intermediate species, such as formate (*HCOO) or carboxyl (*COOH), and they eventually decompose to yield *CO and *OH. As reported in the literature, reducible oxide-supported noble metals follow the associative pathway [12]. For instance, Zhao et al. revealed the size effect of Pt nanoparticles (NP)s on CeO_2_ support towards RWGS reaction [13]. On the basis of the aforementioned discussion, it can be concluded that the RWGS reaction is sensitive to the type of catalyst, and to achieve the optimal CO selectivity and production yield at low temperatures, it is imperative to develop highly efficient catalysts that have two adjacent active sites for CO_2_ activation and H_2_ dissociation. In detail, the catalytic materials with a single reaction site for CO_2_ reduction lead to quick failure due to the severe coke effect, while catalytic materials with single reaction sites for H_2_ dissociation are inactive towards CO_2_. Consequently, potential synergy between two adjacent reaction sites (for CO_2_ activation and H_2_ dissociation) is desired for optimum product yield. For instance, our previous study demonstrated the potential collaboration between neighbouring reaction sites of tetrahedral symmetric nickel oxide (NiO_T_) and Pd in bimetallic NiO_T_Pd-T nanocatalysts, where NiO_T_ and Pd synergistically trigger the CO_2_ activation and H_2_ dissociation, respectively [14]. In addition, the previously published literature reported that surface O^V^ sites in the catalyst supports as well as in the active metal can boost the CO_2_ activation step during CO_2_ hydrogenation, and, thus, the catalytic performance can be improved [15,16]. The aforementioned arguments suggest that the surface atomic configuration is the cornerstone for catalytic activity; therefore, exploration of next-generation techniques to manipulate the surface atomic arrangement is important. In this context, thermal annealing has been frequently employed for controlling the heteroatomic intermixing on the surface as well as the subsurface domains of nanocatalysts for improving the catalytic performance in various reactions; nonetheless, the longer operational time and high energy input make it highly reluctant to use on a commercial scale [17]. In contrast, pulsed laser irradiation with controllable duration and high energy photon flux has emerged as a potential technique for desired atomic manipulation to design the material with optimum functionality [18]. Moreover, this method enables the opportunity to restructure the nanocatalyst surface within a sub-nanometer range, promoting strong heteroatomic intermixing and enhancing catalytic activity. In our previous study, multiple metal-to-metal oxide heterogeneous interfaces have been formed in a trimetallic system (CuNiPd) for enhanced CO_2_ reduction performance by using sub-millisecond laser annealing [19]. 

By keeping the aforementioned scenarios in view, herein, we have fabricated Pd NPs on the cobalt oxide support (hereafter denoted as CoPd) for high-performance RWGS reactions. Furthermore, a pulsed laser beam with per-pulse energies of 1 mJ and 10 mJ was used for a fixed duration of 10 s to manipulate the surface and/or sub-surface atomic arrangements of as-prepared CoPd nanocatalyst to improve the catalytic performance towards RWGS reaction. For the optimum case, when the per-pulse energy of the pulsed laser beam was 10 mJ (hereafter denoted as CoPd-10), some cobalt oxide (CoOx) atoms migrated on the surface of Pd nanoparticles (defect sites of Pd). With such an atomic rearrangement, the CoPd-10 nanocatalyst delivered the CO production yield of ~1667 μmol g^−1^_catalyst_, with CO selectivity of ~88% at 573 K, which was enhanced 41% as compared with pristine CoPd (~976 μmol g^−1^_catalyst_). The results of the physical investigations and electrochemical analysis indicated that the potential synergism between surface-anchored atomic CoOx species and adjacent Pd active sites boosted the CO production yield of the CoPd-10 nanocatalyst, where CoOx and Pd reaction sites simultaneously promoted the CO_2_ activation and H_2_ splitting. We envision that the obtained results could serve as a basis for developing catalysts with improved activity and selectivity for the RWGS reaction.

## 2. Experimental Section

### 2.1. Materials and Methods

The CoPd nanocatalysts were prepared by a sequential and vigorous wet chemical reduction method. To improve the metal–support interaction and achieve better dispersion, before synthesis, the surface functionalization of the catalyst support (i.e., carbon black (UR-XC72, UniRegion Bio-Tech, Palo Alto, CA, USA)) was achieved via acid treatment. [20] Subsequently, in the first step, 3 g of 2 wt.% acid-treated carbon black (hereafter denoted as active carbon (AC)) solution (i.e., 60 mg of AC) was dispersed in 3.06 g of 0.1 M cobalt (III) chloride (99%, Sigma-Aldrich Co., St. Louis, MO, USA) (i.e., the weight ratio of Co to AC was 30 wt.%) and stirred at 600 rpm for 6 h at room temperature (solution A). In the second step, 0.01 g of sodium borohydride (NaBH_4_; 99%, Sigma-Aldrich Co.) in 5 mL of D.I. water was instantly dropped to solution A (i.e., Co^3+^ ions adsorbed on AC surface) to reduce the Co^3+^ ions on the AC surface (i.e., formation of Co-AC) (solution B). Finally, the, 3.06 g of a palladium (Pd) precursor solution (i.e., 0.1 M solution containing 0.306 mmol of Pd metal ions (PdCl_2_, 99%, Sigma-Aldrich Co.)) was added to solution B. In this step, the Pd^2+^ ions were reduced on CoOx support via NaBH_4_ added in the second step (the amount of NaBH_4_ in the second step was measured for reducing both Co^3+^ and Pd^2+^ ions), and the CoPd nanocatalyst was formed. The final product was sequentially washed with acetone, isopropyl alcohol (IPA), and DI water several times, then dried at 70 °C for 24 h. As-prepared CoPd nanocatalyst was further subjected to sub-millisecond pulsed laser irradiation with per-pulse energies of 1 mJ and 10 mJ to restructure the surface of the material. Hereafter, the CoPd nanocatalysts irradiated with 1 mJ and 10 mJ per-pulse energies are denoted as CoPd-1 and CoPd-10, respectively. In this study, a laser beam with the wavelength of 976 nm and 850 μs pulses was generated from the diode laser. Further details of laser setup are provided in supplementary information.

### 2.2. Physical Characterizations

The structural characteristics of as-prepared CoPd nanocatalysts were examined by cross-referencing the results of high-resolution transmission electron microscopy (HRTEM), X-ray diffraction (XRD), X-ray absorption spectroscopy (XAS), and X-ray photoelectron spectroscopy (XPS). The HRTEM images were collected at National Tsing Hua University, Taiwan. The XRD patterns were obtained at the beamline of BL-01C2 in the National Synchrotron Radiation Research Center (NSRRC), where the wavelength of incident X-rays was 0.688 Å (18 keV). The XAS was carried out at beamlines BL-17C and 01C1 in NSRRC, Taiwan, whereas the XPS spectra were measured at beamline BL-24A1 in NSRRC, Taiwan.

### 2.3. Electrochemical Characterizations and Gaseous Product Analysis

The CO-stripping voltammograms were collected using a three-electrode system, where a glassy carbon electrode, Pt wire, and Ag/AgCl electrode were used as the working, counter, and reference electrodes, respectively. The adsorption of CO on the surface of the catalyst was performed by purging CO into 0.5 M H_2_SO_4_ at 0.05 V (vs. RHE) for 20 min. Then, the CO stripping voltammetry was measured between −0.10 and 1.20 V (vs. RHE) in N_2_-saturated 0.5 M H_2_SO_4_ solution at a scan rate of 50 mVs^−1^. Finally, the catalytic performances of CoPd nanocatalysts towards CO_2_ conversion were evaluated by using the previously reported protocol [14,19].

## 3. Results and Discussion

### Structural Properties of CoPd Nanocatalysts

**Figure 1:**
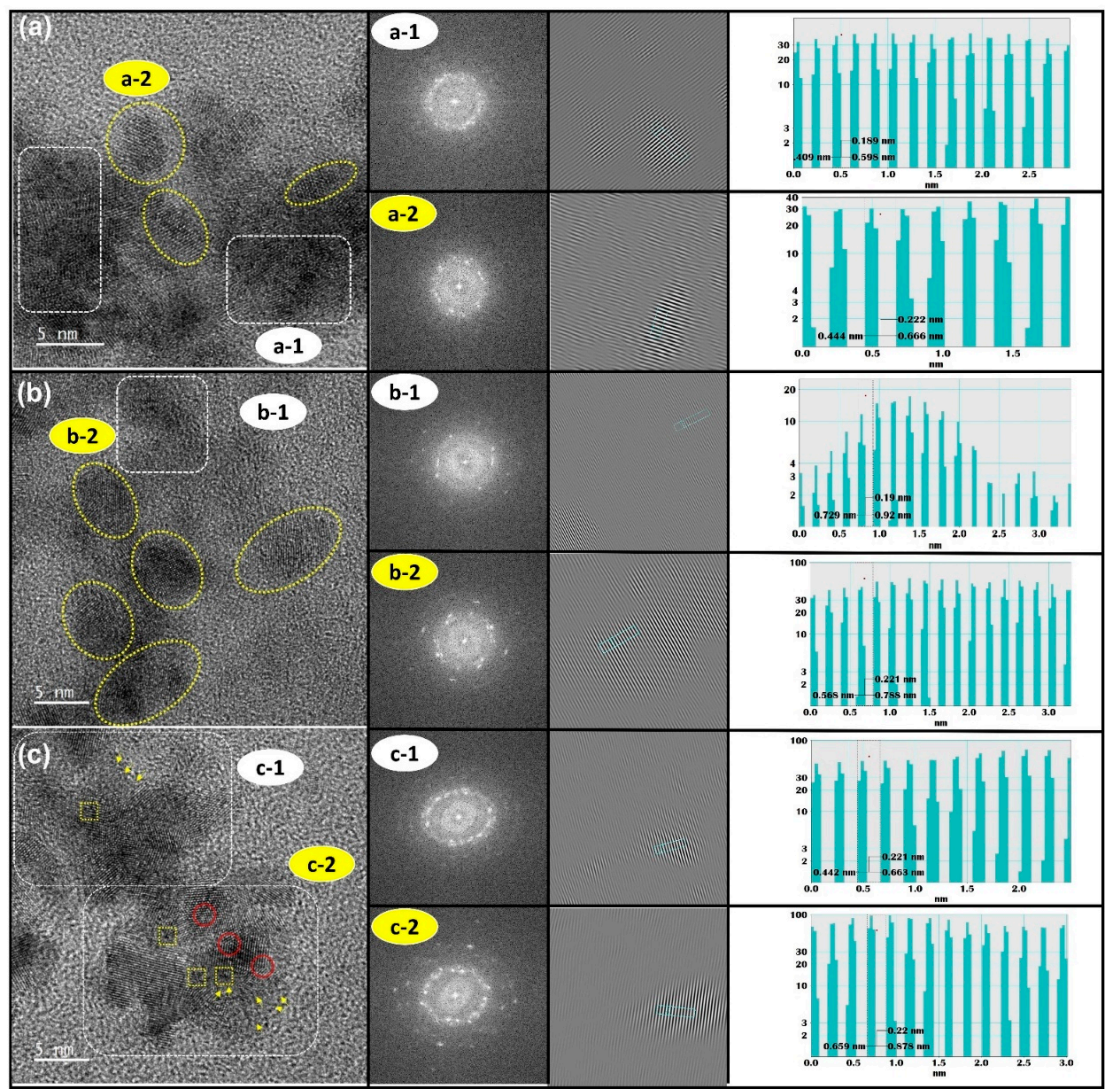
HRTEM images of (**a**) pristine CoPd, (**b**) CoPd-1, and (**c**) CoPd-10. The forward Fourier transformation (FFT) pattern of the selected area in HRTEM images is shown in the insets. The d-spacing values of experimental samples are calculated by using inverse Fourier transform (IFT) and their corresponding line histograms (insets).

The crystal structure and surface atomic arrangements of pristine CoPd and laser-irradiated CoPd nanocatalysts were revealed by HRTEM. Figure 1a shows the HRTEM image of the pristine CoPd nanocatalyst. Accordingly, the majority of Pd domains were covered by a thin layer of amorphous CoOx (denoted in a white square; region (a-1)), while the minority of the Pd domains were exposed to the surface (denoted by yellow circles; region (a-2)). Such characteristics were obvious due to the high extent of galvanic replacement reaction between the Co atoms and Pd^2+^ ions (Co + Pd^2+^ → Co^3+^ + Pd^0^), followed by redeposition of the residual Pd ^2+^ and Co^3+^ ions [21]. These observations were further confirmed by the Fourier transformation (FFT), inverse Fourier transform (IFT) patterns, and their corresponding line histograms, where ring-like FFT patterns in inner lattices and fuzzy patterns in outer space (a-1), respectively, corresponded to the existence of polycrystalline Pd NPs covered by amorphous CoOx [22]. In addition, symmetrically aligned bright spots in the FFT pattern of the region (a-2) suggested the formation of locally ordered Pd NPs. Moreover, the line histograms determined that the interlayer (d)-spacing of regions (a-1) and (a-2) were 0.189 and 0.222, respectively, which corresponded to the Co_3_O_4_ (130) (mp-1271793) and Pd (111), which is in good agreement with the aforementioned observations. Furthermore, a significant surface restructure was observed when the CoPd nanocatalyst was exposed to the laser with 1 mJ per-pulse energy (i.e., CoPd-1). As shown in Figure 1b, the majority of the Pd domains were exposed to the surface (denoted by yellow circles; region (b-2)) and could be attributed to the removal of the surface amorphous CoOx layer due to laser irradiation. However, some of the amorphous CoOx domains were still present on the surface (denoted in a white square; region (b-1)) due to limited per-pulse energy (i.e., 1 mJ). These scenarios were cross-referenced by the FFT patterns and line-histogram-determined d-spacing, where symmetrical aligned bright spots in both regions indicated the formation of long-range ordered structures due to the removal of surface oxide. Further raising the per-pulse energy to 10 mJ led to the formation of a completely different nanoarchitecture for the CoPd-10 nanocatalyst (Figure 1c), where the surface oxide layer was completely removed, and long-range ordered Pd NPs with twin boundaries (denoted by red circles) were formed on the surface. Moreover, the higher per-pulse energy (i.e., 10 mJ) induced a high extent of atomic migration; therefore, some of the Co-atoms from the CoOx support were deposited in the defect sites of Pd NPs (denoted by yellow squares). Meanwhile, the yellow arrows denotes surface defects of Pd NPs. In this way, atomic CoOx-species-decorated Pd NPs were formed on the CoOx support underneath. These scenarios were further confirmed by FFT patterns and d-spacing values, where similar FFT patterns and d-spacing values were observed on the whole surface, confirming the uniform distribution of decorated CoOx species.

**Figure 2:**
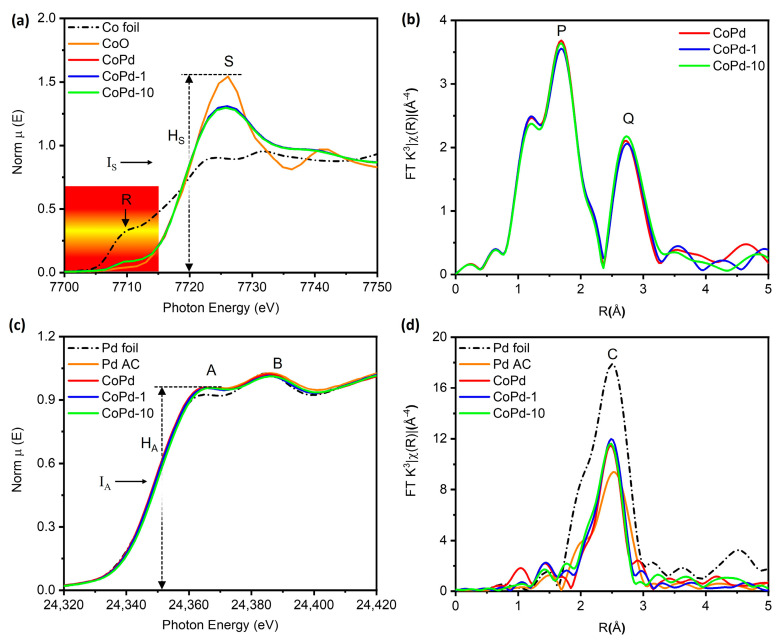
X-ray absorption spectroscopy of the experimental samples compared with those of reference samples. (**a**) XANES and (**b**) FT-EXAFS spectra of the experimental samples at the Co K-edge. (**c**) XANES and (**d**) FT-EXAFS spectra of the experimental samples at the Pd K-edge.

The effect of laser irradiation with different per-pulse energies on the local atomic and electronic properties of CoPd nanocatalyst was explored by cross-referencing the results of the Co K-edge and Pd K-edge XAS analyses. Figure 2a shows the X-ray absorption near-edge spectroscopy (XANES) spectra of pristine and laser-irradiated CoPd nanocatalysts, while the XANES spectra of standard Co foil and CoO were compared for reference. The XANES spectra at Co K-edge showed three main regions, including the pre-edge “R”, the position of the inflection point (I_S_), and the intensity of the absorption edge (or whiteline) (H_S_), which, respectively, corresponded to the local geometry around the Co atoms, the oxidation state, and the extent of occupied/unoccupied state (due to electronic interaction with neighbouring atoms) of the targeting atom [23,24]. As shown in Figure 2a, the CoPd, CoPd-1, and CoPd-10 nanocatalysts exhibited similar features in all three regions, suggesting the unchanged local atomic and electronic properties of CoOx in CoPd after laser irradiation. In addition, the inflection point position of experimental nanocatalysts, similar to that of CoO, implied that the Co was present in the form of CoO in all samples. Figure 2b shows the Fourier-transformed extended X-ray absorption fine structure (FT-EXAFS) spectra of experimental samples at Co K-edge; the corresponding structural parameters are summarized in Table 1, and the fitting curves are shown in Figure S1. The peaks “P” and ”Q” in Figure 2b correspond to the Co-O and Co-Co/Pd bond pairs. Meanwhile, as listed in Table 1, the Co-O bond pairs exhibited a higher coordination number (CN) as compared with the Co-Co and Co-Pd bond pairs, confirming that the Co was present in oxidized form, which is in good agreement with the aforementioned results. An even closer inspection of model-simulated fitting results revealed that CoPd-10 exhibited the highest CN for the Co-O bond pair (CN_Co-O_ = 2.84), suggesting that Co atoms were exposed to the surface at the highest extent. These scenarios are in good agreement with the HRTEM results, where atomic CoOx species were observed on the surface of Pd NPs. Furthermore, Figure 2c shows the XANES spectra of experimental samples compared with the Pd foil and AC-supported Pd NPs, where peaks A and B correspond to the 1s → 5p–4f electron transitions [25,26]. Accordingly, the inflection point position of the experimental samples, similar to that of the Pd foil, confirmed that Pd was present in metallic form. Moreover, the lowest white-line intensities (H_A_ and H_B_) implied the lowest empty state in Pd 5p/4f orbitals due to the highest extent of electron relocation from Co to Pd in the CoPd-10 nanocatalyst [27]. Figure 2d shows the FT-EXAFS spectra of experimental samples at Pd K-edge; the fitting parameters are summarized in Table 1, and the fitting curves are shown in Figure S2. Accordingly, nearly similar CN for Pd-O, Pd-Co, and Pd-Pd bond pairs indicateed that the Pd domains were less affected by laser irradiation. However, the lowest radial distance for the Pd-Co bond pair (R_Pd-Co_ = 3.102) again confirmed that the CoOx species were decorated in the defect sites of Pd NPs in CoPd-10. Moreover, the CN for the Pd-O bond pair was higher for CoPd-1 and CoPd-10 as compared with pristine CoPd, indicating a certain extent of Co oxide anchored on the metallic Pd defect sites.

**Figure 3:**
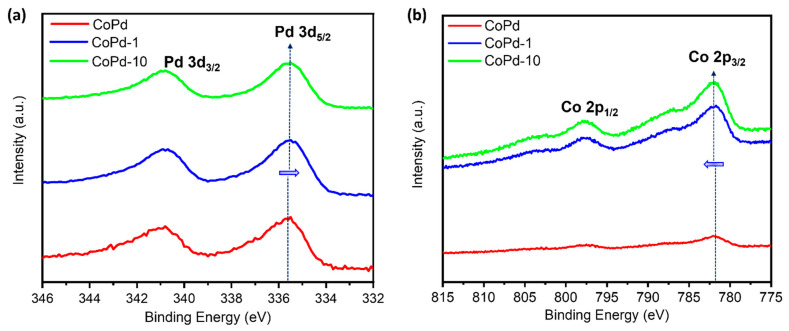
X-ray photoelectron spectroscopy of experimental samples at (**a**) Pd-3d and (**b**) Co-2p orbitals.

To gain more insight into the electronic interaction between the Co and Pd domains and to elucidate the binding energies of the constituting elements, the XPS was employed. Figure 3a,b show the comparative XPS spectra of experimental samples at Pd-3d and Co-2p orbitals. As shown in Figure 3a, the doublet peaks at ~335.6 eV and ~340.9 eV, respectively, corresponded to the Pd-3d_5/2_ and Pd-3d_3/2_ orbitals, where the lower binding energy was observed for CoPd-1 and CoPd-10 samples at Pd-3d orbitals as compared with pristine CoPd. Such characteristics suggest to some extent the electron relocation from Co-to-Pd atoms and are consistent with the aforementioned Pd K-edge XAS analysis. The results of XPS analysis at the Co-2p core level (Figure 3b) confirmed these scenarios, where the CoPd-1 and CoPd-10 exhibited higher binding energy at Co-2p orbitals as compared with pristine CoPd. These results integrally confirmed the electron localization from Co-to Pd atoms in laser-irradiated samples. 

**Figure 4:**
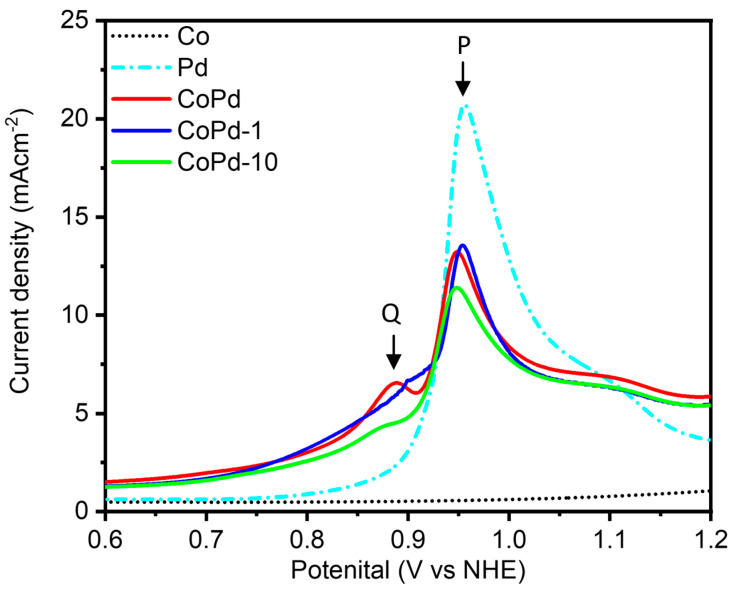
CO-stripping curves of as-prepared CoPd, CoPd-1, and CoPd-10 nanocatalysts compared with control samples (Co-AC and Pd-AC).

The CO-stripping analysis was utilized to explain the effect of laser irradiation on the surface chemical identities of the CoPd nanocatalyst. Figure 4 shows the CO stripping curves of CoPd, CoPd-1, and CoPd-10 samples compared with the control samples (Pd-AC and Co-AC). Accordingly, the nearly flattened current responses for AC-supported Co NPs (i.e., the Co-AC) suggested their inert behaviour towards CO molecules [23]. Meanwhile, the AC-supported Pd NPs (i.e., Pd-AC) exhibited a sharp CO-oxidation peak (P) at ~0.955 V vs. NHE, which corresponded to the CO-oxidation from the closely packed (111) facet. However, the absence of CO-oxidation peaks at lower potentials suggested that AC-supported Pd NPs had less selectivity between open and closed facets for Co-oxidation. For pristine CoPd, a slight offset of the main CO oxidation peak P indicated the reduced energy barrier for CO oxidation as compared with Pd-AC and could be attributed to some extent to the electron localization from Co-to-Pd atoms due to the electronegativity difference and lattice mismatch (consistently conformed with the XPS spectra of pristine CoPd (Figure S3)). Moreover, the presence of an additional peak Q referred to the CO oxidation at the low energy barrier reaction sites (e.g., heterogenous Co-Pd interface or CoPd alloys due to the limited extent of heteroatomic intermixing). Meanwhile, the suppression of the CO-oxidation peak could be attributed to the surface coverage of Pd sites by a thin layer of amorphous CoOx, as consistently proved by the flattened CO-stripping curve of Co-AC (i.e., inert behaviour of CoO_x_ towards CO molecules). For CoPd-1 and CoPd-10 samples, the absence of a peak (Q) suggested the removal of the surface oxide layer after laser irradiation. Such scenarios were further confirmed by the increased intensity of the CO-oxidation peak for CoPd-1, where very less atomic CoOx species were decorated on the surface of Pd NPs due to limited atomic migration (less per-pulse energy of 1 mJ). On the other hand, the CoPd-10 sample showed a CO-oxidation peak at the lowest potential as compared with the CoPd and CoPd-1 nanocatalysts, which could be attributed to the highest extent of electron relocation from Co-to-Pd atoms and is in good agreement with the XAS and XPS results. Meanwhile, the suppressed peak intensity of CoPd-10 as compared with CoPd-1 corresponded to the high density of atomic CoOx species on the surface of Pd NPs due to a high per-pulse energy and, thus, a higher extent of atomic migration.

**Scheme 1:**
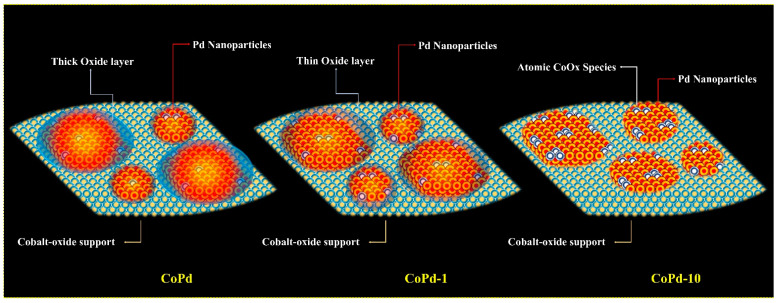
The schematic representation for the atomic structures of CoPd, CoPd-1, and CoPd-10 nanocatalysts.

By cross-referencing the outcomes of the aforementioned physical and electrochemical characterization, the atomic structures of pristine CoPd, CoPd-1, and CoPd-10 nanocatalysts were proposed and are shown in Scheme 1. Accordingly, the pristine CoPd nanocatalyst comprised the CoOx-supported Pd nanoparticles with a thick oxide layer on the surface due to the high extent of galvanic replacement reaction between Co atoms and Pd^2+^ ions (Co + Pd^2+^ → Co^3+^ + Pd^0^), followed by redeposition of residual Pd ^2+^ and Co^3+^ ions. Furthermore, the CoPd nanocatalyst was subjected to pulsed laser irradiation with the per-pulse energy of 1 mJ (i.e., CoPd-1). In this case, due to the relatively lower per-pulse energy of 1 mJ, the atomic migration was limited; thus, the CoOx species was deposited in the defect sites of Pd NPs to a lesser extent, while an unconformable thin oxide layer still existed on the surface of the CoPd-1 nanocatalyst. When the per-pulse energy was increased to 10 mJ, the surface oxide layer was completely removed from the surface of the CoPd-10 nanocatalyst, and a high density of atomic CoOx species were anchored in the defect sites as well as on the surface of the Pd NPs.

**Figure 5:**
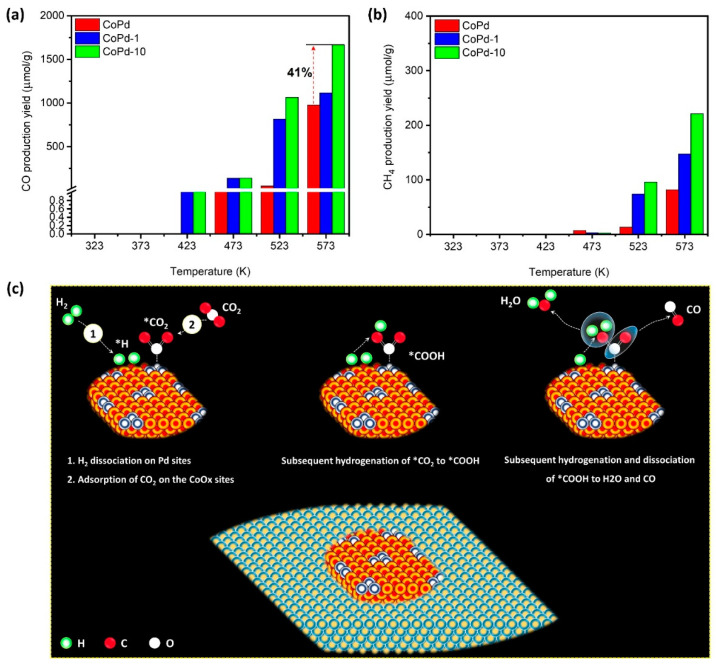
Gas chromatography (GC)-determined CO_2_ reduction reaction results for the experimental samples. (**a**) CO and (**b**) CH_4_ production yield in reaction gas (CO_2_ + 3H_2_). (**c**) The plausible reaction pathways of RWGS reaction on CoPd-10 nanocatalyst.

The catalytic performances of experimental nanocatalysts were evaluated across the temperature range from room temperature (RT) to 573 K at ambient pressure in a gas chromatography (GC) system equipped with a PDHID detector under a flowing reaction gas of H_2_/CO_2_ (3/1). Figure 5a shows the CO production yield of experimental nanocatalysts in the reaction gas of CO_2_:H_2_ = 1:3. Accordingly, the pristine CoPd nanocatalyst was chemically inert toward CO_2_ until reaching a temperature of 423 K, while the CO production yields for laser-irradiated CoPd-1 and CoPd-10 nanocatalysts were 6.70 μmol g^−1^_catalyst_ and 7.01 μmol g^−1^_catalyst_, respectively, at 423 K, suggesting the laser-irradiation-mediated surface restructure successfully decreased the onset temperature of the CoPd nanocatalyst for CO production by 50 °C. The laser-irradiated CoPd-1 and CoPd-10 nanocatalysts exhibited higher CO production yields across the temperature range as compared with the pristine CoPd, where the CoPd-10 nanocatalyst achieved the highest CO production yield of ∼1667 μmol g^−1^_catalyst_ at 573 K. This value reflected improvements of ∼41% and ∼33% as compared with the pristine CoPd (∼976 μmol g^−1^_catalyst_) and CoPd-1 (∼1114 μmol g^−1^_catalyst_) nanocatalysts, respectively. In addition, the CoPd-10 nanocatalyst attained CO selectivity as high as ∼80%. Such a high catalytic performance at high temperature range was obvious because H_2_ splitting on Pd reaction sites increased at high temperatures. In this case, the hydrogenation rate of adsorbed CO_2_ (i.e., formation of *COOH) increased, thus increasing the CO production yield. The results of physical characterizations along with the electrochemical analysis suggested that such an improved catalytic performance of CoPd-10 nanocatalyst originated from the synergistic cooperation between the surface-anchored atomic CoOx species and adjacent Pd domains. Thus, the Pd reaction sites were favourable for H_2_ splitting, while adjacent atomic-scale metal oxide species with possible oxygen vacancies promoted CO_2_ adsorption, followed by reduction. [28] Therefore, it can be concluded that the Pd and adjacent atomic scale CoOx reaction sites synergistically triggered the H_2_ dissociation and CO_2_ activation steps during the CO_2_ reduction reaction (CO_2_RR). Furthermore, as shown in Figure 5b, the experimental nanocatalysts showed a much lower CH_4_ production yield as compared with the CO production yield in the reaction gas, which confirmed the suppressed competitive CO_2_ methanation process, and, therefore, the high CO-selectivity was achieved. The plausible reaction mechanism of the RWGS reaction on the surface of the CoPd-10 nanocatalyst is shown in Figure 5c.

## 4. Conclusions

Rational manipulation of surface atomic arrangements is a cardinal performance-determining factor for heterogeneous catalysts. Herein, we used a sub-millisecond pulsed laser annealing technique with different per-pulse energies to manipulate the surface configuration of cobalt oxide supported Pd (CoPd) nanoparticles for enhanced reverse water–gas shift (RWGS) reaction. For the optimum case, when the per-pulse energy was 10 mJ, the CoPd-10 nanocatalyst exhibited the highest CO production yield of ∼1667 μmol g^−1^_catalyst_ at 573 K, with the CO selectivity as high as ∼80%. This CO production yield was enhanced ∼41% and ∼33% as compared with the pristine CoPd (∼976 μmol g^−1^_catalyst_) and CoPd-1 (∼1114 μmol g^−1^_catalyst_) nanocatalysts, respectively. The results of physical investigations, electrochemical analysis, and gas chromatography (GC) results indicated that the enhanced catalytic activity and selectivity of the CoPd-10 nanocatalyst originated from the potential synergy between surface-anchored atomic CoOx species and neighbouring Pd reaction sites, which, respectively, promoted the CO_2_ activation and H_2_ splitting. Briefly stated, the obtained results are expected to mark a step ahead in designing high-performance nanocatalysts for various redox reactions by using the pulsed laser technique.

## Figures and Tables

**Table 1:** Quantitative results of X-ray absorption spectroscopy model analysis at Co and Pd K-edges of experimental samples.

Sample	Co K-Edge			Pd K-Edge		
	Bond Pair	CN	R	Bond Pair	CN	R
**CoPd**	Co-O	2.636	2.029	Pd-O	0.89	2.046
	Co-Co	0.92	2.052	Pd-Pd	5.236	2.742
	Co-Pd	0.591	3.044	Pd-Co	0.788	3.280
**CoPd-1**	Co-O	2.70	2.016	Pd-O	0.98	2.301
	Co-Co	1.58	3.093	Pd-Pd	5.02	2.745
	Co-Pd	0.685	3.133	Pd-Co	0.665	3.224
**CoPd-10**	Co-O	2.84	2.033	Pd-O	0.94	2.107
	Co-Co	1.803	2.949	Pd-Pd	5.16	2.746
	Co-Pd	0.650	3.186	Pd-Co	0.572	3.102
**Pd-AC**	N/A			Pd-Pd	4.96	2.741

## Data Availability

The data presented in this study are available on request from the corresponding author.

## Supplementary Materials

The following supporting information can be downloaded at: https://www.mdpi.com/article/10.3390/nano13111801/s1. Figure S1. Model analysis fitting curves compared with experimental FT-EXAFS spectra at Co K-edge of (a) CoPd, (b) CoPd-1, and (c) CoPd-10; Figure S2. Model analysis fitting curves compared with experimental FT-EXAFS spectra at Pd K-edge of (a) Pd AC, (b) CoPd, (c) CoPd-1, and (d) CoPd-10; Figure S3. X-ray photoelectron spectroscopy of experimental NCs at Pd 3D orbital of CoPd NC with Pd/AC.
