# Supplementary material for: Sub-Millisecond Laser-Irradiation-Mediated Surface Restructure Boosts the CO Production Yield of Cobalt Oxide Supported Pd Nanoparticles
Source: Nanomaterials (Basel). 2023 Jun 5;13(11):1801. doi: 10.3390/nano13111801 (PMC10255247; doi:10.3390/nano13111801)
Supplement: Supplementary file 1 [file nanomaterials-13-01801-s001.zip › nanomaterials-2424388-supplementary.pdf]

nanomaterials

MDPI

Supporting Information

Sub-Millisecond Laser-Irradiation-Mediated Surface Restructure Boosts the CO Production Yield of Cobalt Oxide Supported Pd Nanoparticles

Praveen Kumar Saravanan,[a] Dinesh Bhalothia,[b†*] Guo-Heng Huang,[b] Amisha Beniwal,[b] Mingxing Cheng,[b,c] Yu-Chieh Chao,[b,d] Ming-Wei Lin,[b,d] Po-Chun Chen,[a*] Tsan-Yao Chen[b,e*]

[a.] Department of Materials and Mineral Resources Engineering, National Taipei University of Technology, Taipei 10608, Taiwan

[b.] Department of Engineering and System Science, National Tsing Hua University, Hsinchu 30013, Taiwan

[c.] Department of Electrical Engineering and Electronics, University of Liverpool, Liverpool L69 3GJ, UK

[d.] Institute of Nuclear Engineering and Science, National Tsing Hua University, Hsinchu 30013, Taiwan

[e.] Hierarchical Green-Energy Materials (Hi-GEM) Research Centre, National Cheng Kung University, Tainan 70101, Taiwan

1. Details of Laser Annealing Setup

The laser annealing setup is primarily composed of the diode laser (Model PM25, Lastronics GmbH), sets of imaging lenses, and the sample holder. In this study, an 850 μs pulse laser with 976 nm is generated from the diode laser. The sample holder with a diameter of 10 mm is fabricated by sinking a portion of a frosted glass plate to a depth of 3 mm, which can be filled with 2 mg of catalysts for each annealing operation. Under the fixed operating time of 10 s and the total beam energy of 1 J, repetition rates of 10 Hz and 100 Hz are assigned for the laser pulses to irradiate the sample with single pulse energies of 10 mJ and 1 mJ, respectively; in this way, each 850 μs, 10 mJ pulse provides a laser power of 11.77 W and intensity of 5.46 $J/cm^2/s$ for annealing operation at 10 Hz, while the laser power and intensity reduce to 1.18 W and 0.55 $J/cm^2/s$, respectively, when using 850 μs, 1 mJ pulses to anneal samples at 100 Hz.

2. Model analysis fitting curves compared with experimental FT-EXAFS spectra at Co K-edge.

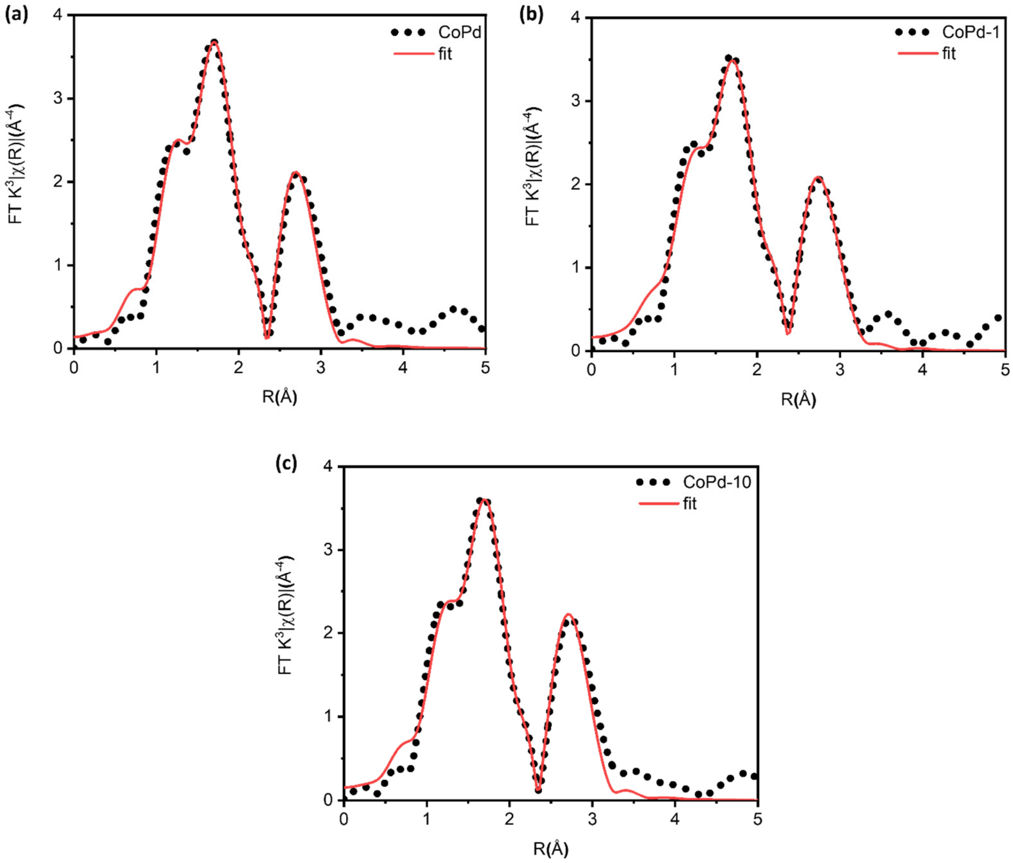

Figure S1. Model analysis fitting curves compared with experimental FT-EXAFS spectra at Co K-edge of (a) CoPd, (b) CoPd-1, and (c) CoPd-10.

3. Model analysis fitting curves compared with experimental FT-EXAFS spectra at Pd K-edge.

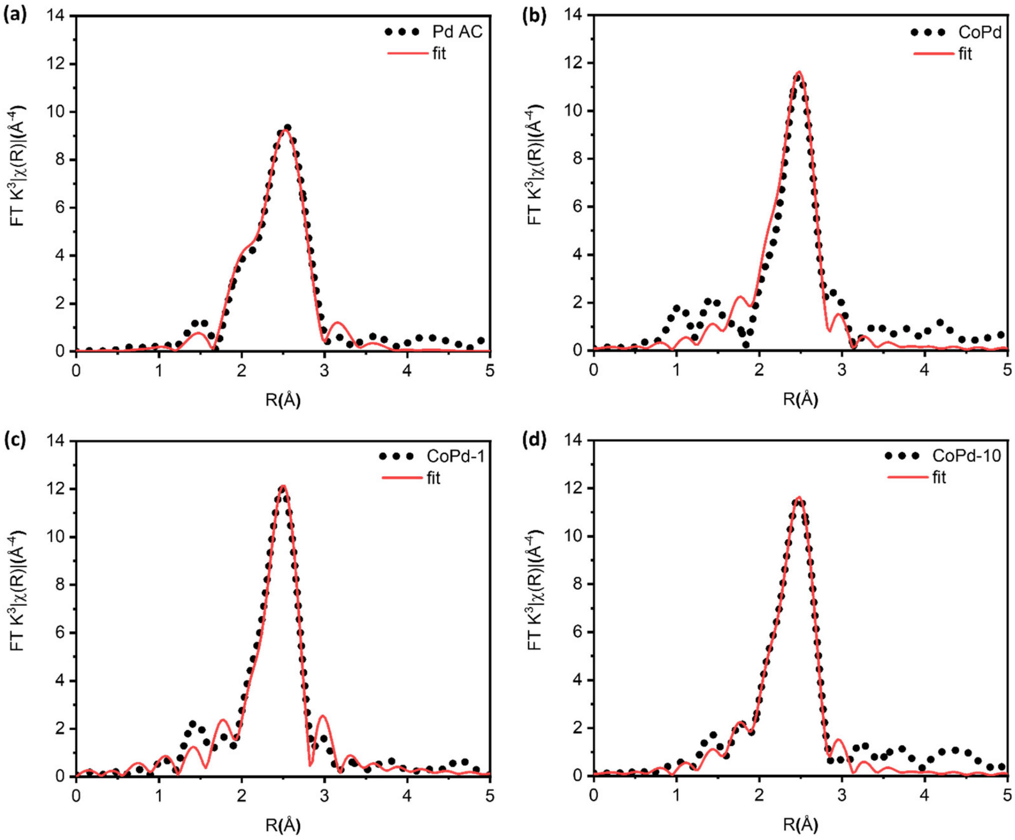

Figure S2. Model analysis fitting curves compared with experimental FT-EXAFS spectra at Pd K-edge of (a) Pd AC, (b) CoPd, (c) CoPd-1, and (d) CoPd-10.

4. X-ray photoelectron spectroscopy of CoPd with Pd-AC.

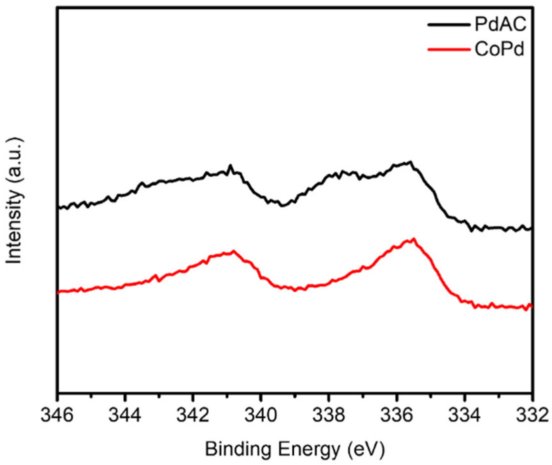

Figure S3. X-ray photoelectron spectroscopy of experimental NCs at Pd 3D orbital of CoPd NC with Pd/AC.
